# Temporal processing of self-motion: modeling reaction times for rotations and translations

**DOI:** 10.1007/s00221-013-3536-y

**Published:** 2013-05-12

**Authors:** Florian Soyka, Heinrich H. Bülthoff, Michael Barnett-Cowan

**Affiliations:** 1 Department of Human Perception, Cognition and Action, Max Planck Institute for Biological Cybernetics, Spemannstraße 38, 72076 Tübingen, Germany; 2 Department of Brain and Cognitive Engineering, Korea University, Anamdong, Seongbuk-gu, Seoul, 136-713 Korea; 3 Department of Psychology, The Brain and Mind Institute, The University of Western Ontario, London, ON N6A 5B7 Canada

**Keywords:** Reaction time, Self-motion, Perception threshold, Time perception, Latency, Vestibular

## Abstract

In this paper, we show that differences in reaction times (RT) to self-motion depend not only on the duration of the profile, but also on the actual time course of the acceleration. We previously proposed models that described direction discrimination thresholds for rotational and translational motions based on the dynamics of the vestibular sensory organs (otoliths and semi-circular canals). As these models have the potential to describe RT for different motion profiles (e.g., trapezoidal versus triangular acceleration profiles or varying profile durations), we validated these models by measuring RTs in human observers for a direction discrimination task using both translational and rotational motions varying in amplitude, duration and acceleration profile shape in a within-subjects design. In agreement with previous studies, amplitude and duration were found to affect RT, and importantly, we found an influence of the profile shape on RT. The models are able to fit the measured RTs with an accuracy of around 5 ms, and the best-fitting parameters are similar to those found from identifying the models based on threshold measurements. This confirms the validity of the modeling approach and links perceptual thresholds to RT. By establishing a link between vestibular thresholds for self-motion and RT, we show for the first time that RTs to purely inertial motion stimuli can be used as an alternative to threshold measurements for identifying self-motion perception models. This is advantageous, since RT tasks are less challenging for participants and make assessment of vestibular function less fatiguing. Further, our results provide strong evidence that the perceived timing of self-motion stimulation is largely influenced by the response dynamics of the vestibular sensory organs.

## Introduction

The ability to model how and when we perceive self-motion has several important implications. Since the perception of passive self-motion in the dark is mainly mediated by the vestibular system (Walsh 1961; Valko et al. 2012), it provides a measure for vestibular function and has potential applications for diagnosing vestibular patients without the necessity to rely on oculomotor recordings (Merfeld et al. 2010). In addition, self-motion perception models are broadly used for calibrating motion simulators in an effort to optimize fidelity in a virtual environment (Borah et al. 1988; Telban and Cardullo 2005; Grant and Lee 2007). Finally, there is an ongoing debate as to whether the dynamics of self-motion perception correspond to the dynamics of motor responses to vestibular stimulation such as the vestibulo-ocular reflex (VOR). Both perception and action processes are determined by sensory signals from the vestibular system; however, differences in the response dynamics for perception and action have been reported and might reflect additional involvement of central processing (Merfeld et al. 2004; Barnett-Cowan et al. 2005; Merfeld et al. 2005a, b; Bertolini et al. 2011, 2012). Despite the importance of being able to model the perceived timing of self-motion, previous efforts have limited power as to date they have been restricted to exposing participants to sinusoidal acceleration profiles of different durations.

In order to assess self-motion perception in humans, we previously measured direction discrimination thresholds for both translational and rotational motions (Soyka et al. 2011, 2012a). Models based on the dynamics of the vestibular sensory signals were introduced which are able to capture the influence of the specific motion stimulus duration and profile shape on thresholds (e.g., threshold differences between sinusoidal or triangular acceleration profiles). These models could also be used to describe reaction times (RT) for discriminating motion directions as a function of varying motion stimuli. The goal of the present study was to measure RTs for different motion profiles and verify whether the model is able to describe the timing of perceived self-motion. To do so, model parameters obtained from previous threshold measurements were compared to estimates obtained from RT measurements in order to test whether the underlying dynamics are similar for threshold and RT measurements.

Establishing a link between thresholds and RTs through modeling is desirable as it could improve upon current methods for assessing self-motion perception. During an RT direction discrimination task in which the stimuli are above threshold, participants’ answers are mostly correct. In contrast, during threshold assessment participants encounter below threshold stimuli and often have to guess the direction of motion. Consequently, threshold tasks can be exhaustive and frustrating to participants. RT tasks, however, are perceived as being easier to perform and are preferred by participants. If one model can describe thresholds and RTs it would then be possible to identify threshold and RT from either measurement.

Our models allow predicting the time it takes a self-motion stimulus to rise above threshold. Note, however, that this is not the total RT. Indeed, the total RT is composed of the time it takes to rise above threshold plus the additional time it takes to cognitively process the sensory signal and the time it takes to press the response button. The models allow calculating the change of the vestibular sensory signal elicited by a motion stimulus and introduce a threshold for the sensory signal. The time it takes for a self-motion stimulus to rise above threshold is predicted by computing when the sensory (neuronal) signal overcomes the threshold. Note that this sensory threshold is different from thresholds reported in terms of motion intensity (e.g., given in terms of peak acceleration). One advantage of looking at the sensory signal is that a single threshold for the sensory signal leads to varying acceleration thresholds depending on the motion stimulus. Therefore, a single neuronal threshold together with a model of the sensory dynamics can describe the behavior of acceleration thresholds for varying motion stimuli.

The present study is unique in that we measured RTs for both translations and rotations using a within-subjects design. Previous RT studies for self-motion perception investigated either rotational motions (Baxter and Travis 1938; Clark and Stewart 1962, 1974; Guedry 1974; Huang and Young 1981) or translational motions (Meiry 1965; Jones and Young 1978; Arrott et al. 1990). To the best of our knowledge, this is the first study to assess the timing of RTs to both rotational and translational motions and to use the same modeling framework to describe both.

Previous efforts to model RTs to self-motion include Mulder (1908), who was the first to report that RTs for rotational motion stimuli—consisting of a step in acceleration—are inversely related to peak acceleration of the stimulus (Mulder’s Law). Thus, the product of peak acceleration and RT are constant. This observation was later explained by assuming that the cupula needs a minimal amount of deflection in order to detect a rotational motion (van Egmond et al. 1949; Guedry 1974). The deflection can be calculated with a torsion-pendulum model (van Egmond et al. 1949). Based on such a model, RTs in response to angular acceleration steps have been successfully described (Rodenburg et al. 1981). A similar approach was used to describe RTs for translational motions as a function of the peak acceleration based on the deflection of the otoconia (Young and Meiry 1968; Jones and Young 1978). These models are similar to ours in the sense that stimulation of the vestibular sensors due to a motion stimulus is calculated, and a minimal stimulation is needed in order to react to the motion. The advantage of our model is that it can deal with arbitrary motion profiles (taking the whole frequency content of the motion into account), whereas previous models were partially restricted to steps in acceleration.

The main goal of this study is to investigate whether RTs can be described based on the same models previously used for describing threshold measurements. If so, this would validate the modeling approach and allow for identifying the model parameters with RTs instead of, or in combination with, threshold measurements which, as discussed above, is advantageous.

## Methods

### Participants

Twenty participants (10 female) took part in the study. They were 20–34 years old (mean = 27 years) and reported no vestibular problems. The participants were paid a standard fee and signed an informed consent form prior to the study. The experiment was conducted in accordance with the requirements of the Helsinki Declaration, and all procedures were reviewed and approved by the ethics committee of the Eberhard Karls Universität Tübingen.

### Motion stimuli

To present motion stimuli to participants, we used the Max Planck Institute CyberMotion Simulator. Further details on its hardware and software specifications are available (Robocoaster, KUKA Roboter GmbH, Germany; Teufel et al. 2007; Robuffo Giordano et al. 2010a, b; Barnett-Cowan et al. 2012a).

**Fig. 1:**
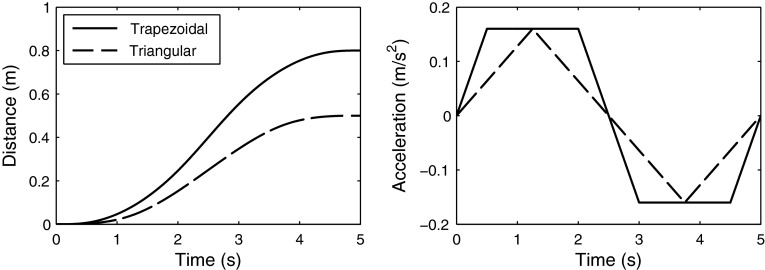
The motion profiles were named after the shape of their acceleration and were either *trapezoidal* or *triangular*. In addition to the profile shapes, durations and amplitudes of the profiles were also varied in order to test their influence on RT

RTs were measured for 8 different conditions (Table 1): 4 translations and 4 head-centered yaw rotations around the earth-vertical body axis. The motion direction of each trial was randomized and was either leftward or rightward rotation or translation. Three motion parameters (duration, amplitude and acceleration profile shape) were varied in order to test the model predictions in various conditions. The profile shape was varied between conditions I–II (V–VI), the duration was varied between conditions II–III (VI–VII) and the amplitude was varied between conditions III–IV (VII–VIII). The conditions were chosen such that every parameter is varied once, while the others are kept constant. The acceleration profile shape was either trapezoidal or triangular (Fig. 1). For the trapezoidal profile, the peak acceleration was reached after *T*/10 s, where *T* is the duration of the profile. Note that amplitudes and thresholds for *rotational* motions are given in terms of velocity and not acceleration (Table 1), because the encoding of the semi-circular canal signal is proportional to velocity (Fernandez and Goldberg 1971), and therefore, rotational motions are usually parameterized in units of velocity. Previous research has made such a distinction of speaking about acceleration when referring to translations (Benson et al. 1986; Soyka et al. 2011) and about velocity when referring to rotations (Benson et al. 1989; Grabherr et al. 2008; Soyka et al. 2012a).

**Table 1:** RTs were measured for 8 conditions: 4 translations and 4 rotations. The profile shape, duration and amplitude were varied. The direction discrimination thresholds were calculated based on previous work (Soyka et al. 2011, 2012a). Note that the profile shape was varied once per motion type (Conditions I–II and V–VI) and then kept constant, while the other parameters (duration and amplitude) were varied

Condition	Type	Profile	Duration	Amplitude	Threshold	Distance
I—Tra 5 s	Translation	Trapezoidal	5 s	0.16 m/s^2^	0.06 m/s^2^	80 cm
II—Tri 5 s	Translation	Triangular	5 s	0.16 m/s^2^	0.07 m/s^2^	50 cm
III—Tri 2.5 s	Translation	Triangular	2.5 s	0.16 m/s^2^	0.06 m/s^2^	12.5 cm
IV—Low Amp	Translation	Triangular	2.5 s	0.09 m/s^2^	0.05 m/s^2^	7 cm
V—Tri 5 s	Rotation	Triangular	5 s	17 °/s	1.6 °/s	42.5°
VI—Tra 5 s	Rotation	Trapezoidal	5 s	17 °/s	1.7 °/s	42.5°
VII—Tra 2.5 s	Rotation	Trapezoidal	2.5 s	17 °/s	1.4 °/s	21.3°
VIII—Low Amp	Rotation	Trapezoidal	2.5 s	10 °/s	1.4 °/s	12.5°

**Fig. 2:**
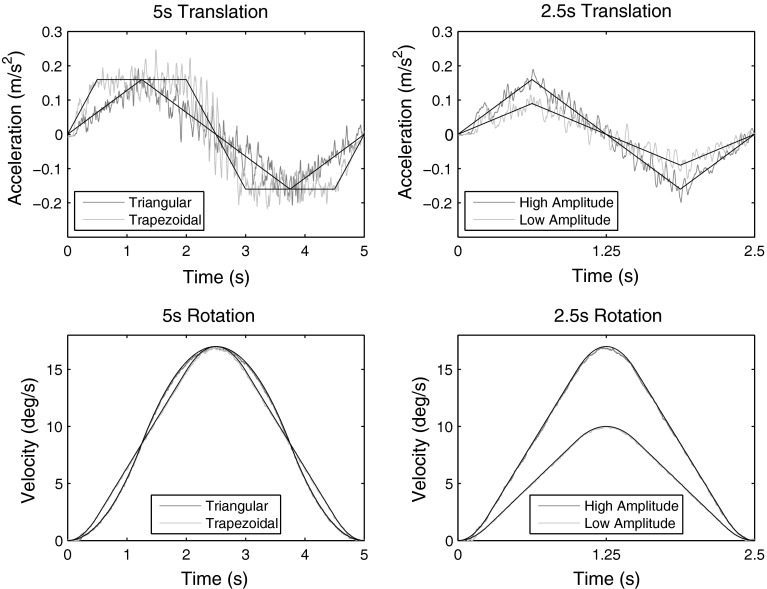
An inertial measurement unit was used to assess the motions produced by the simulator. The measurements are overlaid with the commanded motions such that the cross-correlation between the two signals is maximized. It can be seen that on average, the motions are well reproduced, but that there are high-frequency vibrations for the translational motions

In order to assess the actual motion of the device, an inertial measurement unit (IMU) consisting of three gyroscopes (Analog Devices ADXRS150) and one 3D linear accelerometer (STMicroelectronics LIS3L02AQ) was attached to the seat of the simulator (along the vertical axis specified by the center of the head), and yaw velocity together with linear accelerations were measured at 1,000 Hz. Digital data were obtained from the sensors, and no further filtering was applied. We observed that the IMU measurements deviated from the commanded motion profiles. In order to test if these deviations were random or deterministic, we performed 40 measurements (20 leftward and 20 rightward) per condition and computed the average to reduce any random components (Fig. 2). We chose 40 repetitions since we know from our previous work (Soyka et al. 2012a) that this is a sufficient number of trials to reduce the influence of random vibrations. Translational motions exhibited deterministic high-frequency vibrations, whereas rotational motions had almost no vibrations. To some extent, this is due to the fact that accelerations were measured for translational motions, whereas velocities were measured for rotational motions and are further examined in the Discussion. Note that these vibrations are not random (random vibrations have been averaged out), but occur deterministically during every trial, and therefore, they are part of the motion stimulus. Random vibrations are not an issue (as long as the profiles are still reproduced), since their influence on RTs averages out over many trials. However, the deterministic vibrations have to be taken into account, and therefore, the averaged IMU measurements instead of the commanded motions were used as inputs to the model. Note that this is only possible, because our model can deal with arbitrary motion profiles.

### Experimental procedures

Each participant was tested in all 8 conditions, and a condition lasted until 30 correct responses were given. The duration of each condition was approximately 10 min. After each condition, there was a 5-min break to prevent fatigue. The experiment was divided in 2 blocks, and 4 conditions were tested during each block. Between blocks, there was a 30-min break, such that the total experiment took approximately 2.5 h. Participants were familiarized with the task during an initial training phase consisting of 10 trials. In order to counterbalance possible learning effects, the presentation sequence of the conditions was randomized.

A one-interval two-alternative forced-choice task was used to measure RTs. Participants had to discriminate the direction of motion as fast as possible. In order to reduce the possible influence of internal response criteria and to get similar RTs for all participants, the stimulus amplitudes were above the perceptual threshold (Table 1). This resulted in participants answering correctly in 96 % of all trials, corresponding to approximately 1 erroneous answer per condition. In case they made a mistake, participants were verbally informed about this and the trial was excluded from the analysis. Note that since the stimulus amplitudes were above threshold participants likely were able to indicate their direction of motion during the acceleration phase of the stimulus, not the deceleration phase, in order to make their decision. This is confirmed by our results (Fig. 5) which show that RTs occur before the deceleration phase.

Participants initiated a trial with a button press and the movement began after a constant one-second pause. Note that the duration of the pause is irrelevant for a direction discrimination task. Participants were translated or rotated either leftward or rightward, were instructed to indicate the direction of their motion as fast as possible by pressing one of two buttons and were then moved back to the starting position. Participants were seated in a chair with a 5-point harness and wore light-proof goggles. Acoustic white noise was played during the movements via headphones. Participants wore clothing with long sleeves and trousers, and a fan was directed toward the face to mask possible air movement cues during simulator motion. Between translation trials, there was at least a 1.5-s break (or longer if the participant did not immediately initiate the next trial through a button press). It was recently shown that perceptual thresholds for short fore-aft motions (0.5 s) are influenced by prior translational motions (Crane 2012). These perceptual aftereffects could potentially influence the RTs. Differences between our previous work and the present study are that the intensities of motion stimuli were all above perceptual threshold and stimuli in the present study longer than 0.5 s. Since our study was not designed to test the potential influence of prior motions on RTs, future research is required to assess this possibility. For rotation trials, the break was extended to at least 6 s to avoid perceptual aftereffects (the feeling of a counter rotation) that can occur after rotation in the dark. Participants removed the blindfold during the 6-s break in order to see that they were stationary. This technique is called ‘visual dumping’ and has been shown to shorten the time constant of the post-rotatory nystagmus, which is correlated with perceived rotation (Cohen et al. 1981; Okada et al. 1999). No participant reported feelings of counter rotation.

### Assessing reaction times

The response buttons were connected to the IMU’s digitizer and sampled with the same frequency (1,000 Hz). In order to calculate RTs, the IMU signal was cross-correlated with the commanded motion, which allows for calculating the best estimate of the actual onset of the motion. The difference between the motion onset and the time a button was pressed was taken as RT. Using this method, RT can be measured with an accuracy of 1 ms.

**Fig. 3:**
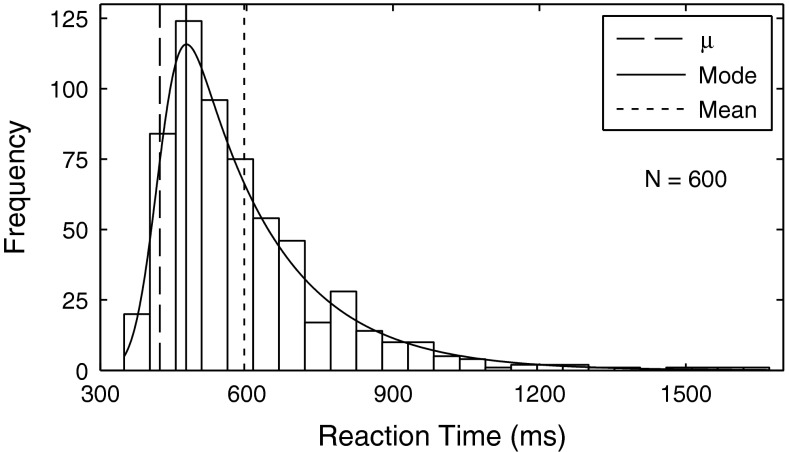
The histogram (25 bins, 53 ms/bin) is based on the RT measurements for condition I of all participants (N = 600). An ex-Gaussian distribution (convolution of a Gaussian and an exponential distribution) was fit to the data. It can be seen that the distribution is skewed and that the mean does not represent a suitable measure. Instead, the parameter *μ* of the ex-Gaussian distribution and the mode are used as measures of the RT

Since RT distributions are not normally distributed but skewed toward longer durations, mean RT is not an appropriate measure of RT (Fig. 3). The mode of the distribution provides a better measure, since it describes the most frequent RT. However, the mode depends on the bin size of the histogram or, alternatively, it can be calculated as the maximum of a specific distribution fitted to the data. It has been shown that a convolution of a Gaussian distribution and an exponential can accurately describe RT distributions (Ratcliff and Murdock 1976; Hockley 1984; Luce 1986). The convolution is called the ex-Gaussian distribution and can be fit to the data with maximum likelihood methods without the need to assume a certain bin size (Ratcliff and Murdock 1976). The equation for the ex-Gaussian distribution is:

1 $$ f(x\left| {\mu ,\sigma ,\tau } \right.) = \frac{1}{\tau }\exp \left( {\frac{\mu }{\tau } + \frac{{\sigma^{2} }}{{2\tau^{2} }} - \frac{x}{\tau }} \right)\Upphi \left( {\frac{{x - \mu - {\raise0.7ex\hbox{${\sigma^{2} }$} \!\mathord{\left/ {\vphantom {{\sigma^{2} } \tau }}\right.\kern-0pt} \!\lower0.7ex\hbox{$\tau $}}}}{\sigma }} \right) $$

where Ф is the cumulative distribution function of the normalized Gaussian distribution, *μ* and *σ* describe the Gaussian distribution and *τ* represents the time constant of the exponential. The Gaussian distribution is assumed to represent the decision process, whereas the exponential part represents the residual latency, for example, the time it takes for the motor action required to provide the response (Luce 1986). Therefore, the mean of the Gaussian distribution is not equal to the mode, but is located earlier in time, since after the decision additional time is required in order to provide the response. Since there is no a priori reason to prefer either the parameter *μ* or the mode of the best-fitting distribution as a measure for RT, we report and analyze both measures. The RTs of all participants were combined and fitted with an ex-Gaussian distribution using MATLAB and the DISTRIB toolbox (Lacouture and Cousineau 2008). The mean (*μ*) and the standard deviation (*σ*) of the Gaussian part and the time constant (*τ*) of the exponential part were estimated for each condition. The ex-Gaussian distribution was fit to the combined data of all participants instead of fitting it to the data of individual participants, as each participant performed only 30 trials resulting in too few data points for a reliable fit. Before combining the data of all participants, we fit ex-Gaussian distributions to individual participants, but obtained unreasonable parameters showing that 30 trials are too few for a reliable fit. Fitting to the combined data also reduces the effect of possible individual differences between participants, which might arise from physiological differences or, on a cognitive level, from differences in internal response criteria.

### Fitting reaction times

We previously introduced models capable of describing direction discrimination thresholds for arbitrary translatory or rotatory motion profiles (Soyka et al. 2011, 2012a). These models calculate a signal akin to the change in firing rate of vestibular neurons that would be elicited by a motion stimulus. For the sake of simplicity, we will refer to this signal as the firing rate, but it should be noted that this signal is not a direct description of the firing rate of vestibular neurons. Rather, the signal is comparable to the average dynamic response of a population of vestibular neurons stimulated by an inertial motion. Transfer functions whose structures are based on the anatomy and physiology of the otoliths and semi-circular canals are used to calculate the signal (Eq. 2):

2 $$ H(s) = K \cdot \frac{{(1 + \tau_{N} s)}}{{(1 + \tau_{1} s)(1 + \tau_{2} s)}} $$

The parameters *K*, *τ*_1_ and *τ*_*N*_, are estimated based on our measurements, whereas the parameter *τ*_2_ is taken from the literature since it describes the behavior at frequencies higher than the ones relevant for our work. For translational motions, *τ*_2_ = 0.016*s* is used and for rotational motions *τ*_2_ = 0.015*s*. Note that this model is based on the structure of the peripheral vestibular sensors and assumes that central neural processing does not alter the form of the transfer function. For further details about the transfer functions, we refer to our previous papers (Soyka et al. 2011, 2012a).

**Fig. 4:**
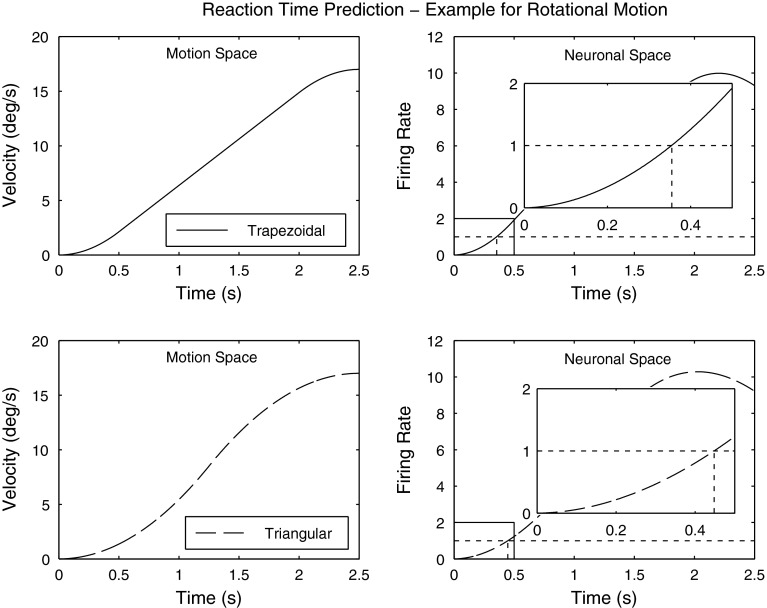
*Left column*: two different rotational motion stimuli. *Right column*: the corresponding change in firing rate in response to the stimuli. The time (*T*_threshold_) it takes for the firing rate to overcome sensory threshold (1 unit of the firing rate) can be found (see *inset*) given the transfer functions of the sensors. *Upper* and *lower* row show the differences between a *trapezoidal* (condition VI) and a *triangular* (condition V) acceleration profile

The main assumption of these models is the existence of a noise level intrinsic to the firing rate. In order to correctly perceive the direction of the motion stimulus, the change in firing rate has to overcome this level of noise (Fig. 4). For a given set of transfer function parameters, this assumption allows us to calculate the motion intensity required in order for the firing rate to overcome the noise level. This intensity represents the threshold prediction of the model for a given motion stimulus and a set of transfer function parameters. Note that threshold is given in terms of the peak acceleration of the motion stimulus for translations or peak velocity for rotations. Since the noise level is unknown, it is arbitrarily fixed at 1 unit of ‘firing rate’, and the models include an additional parameter that inversely scales with the chosen noise level. Given threshold measurements for several motion stimuli varying in duration and profile shape, the parameters of the transfer function can be iteratively varied until a set of best-fitting parameters which locally minimize the error between predictions and measurements is found. Previously, we performed such threshold measurements together with the identification of the best-fitting parameter sets (Soyka et al. 2011, 2012a).

These models can also be used to describe differences in RTs between varying motion stimuli. Given a set of model parameters, it is possible to calculate the time it takes a self-motion stimulus to rise above threshold (Fig. 4). However, this is not the total RT: Indeed, the total RT is composed of the time it takes to rise above threshold (*T*_threshold_) plus the additional time (*T*_additional_) it takes to cognitively process the sensory signal and come to a decision about the direction of the motion. After that, it still takes time to press the response button. Assuming that on average the additional time, *T*_additional_ is constant and independent of the motion stimulus allows calculating meaningful differences in measured RTs (parameter *μ* or mode of the RT distribution) for varying motion stimuli, and thereby eliminating the constant factor *T*_additional_. Therefore, these differences should match differences between the calculated times *T*_threshold_. In order to obtain an optimal model fit, the following error is defined:

3 $$ {\text{error}}_{i,j} = \left( {RT_{i} - RT_{j} } \right) - \left( {T_{{{\text{threshold}},i}} - T_{{{\text{threshold}},j}} } \right) $$

where *i* and *j* denote different conditions. It is possible to form six errors for both translational and rotational motions combining the four different experimental conditions. The error function of the optimization procedure used to find the best-fitting parameters was defined as the sum of the squared errors for the six possible combinations. The transfer function parameters *K*, *τ*_1_ and *τ*_*N*_ were varied until the error function was locally minimized within an error tolerance of 1 (ms)^2^ (‘fminsearch’ function, MATLAB, MathWorks, MA, USA). In this way, two sets of best-fitting transfer function parameters were found for translational and rotational motions, one using the RTs based on the mode and another one using the RT estimates based on the parameter *μ*.

In order to be able to predict the parameter *μ* or the mode of the RT distributions, the constant *T*_additional_ has to be determined and added to the model predictions *T*_threshold_. If the model is able to describe the measurements, an estimate of the constant can be obtained by taking the mean difference between the model predictions *T*_threshold_ and the measured RTs.

## Results

The maximum likelihood parameter estimates *μ*, *σ* and *τ* of the best-fitting ex-Gaussian distribution and the mode of the distribution are reported in Table 2 for each condition. As explained in Ratcliff and Murdock (1976), maximum likelihood estimators have specific asymptotic (large sample size) properties. The estimated parameters are normally distributed, and their variance can be obtained using the inverse of the Fisher information matrix (Wilks 1962). Ratcliff and Murdock (1976) successfully used this approach to characterize parameter estimates for RT distributions similar to those presented here, based on a sample size of *N* = 300. Since our sample size (*N* = 600 per condition) is twice as large as theirs, it is justified to use the same approach in order to calculate standard deviations for the estimated parameters (Table 2). Since the mode is not obtained as part of the maximum likelihood fit there is no estimate of its standard deviation.

**Table 2:** The parameters for the best-fitting ex-Gaussian distribution are given together with an estimate of their standard deviation (in parentheses). Additionally, the mode of the distribution is reported

Condition	Translation				Rotation			
	I	II	III	IV	V	VI	VII	VIII
*μ*, RT [ms]	422 (5)	540 (9)	452 (5)	502 (8)	645 (12)	557 (9)	406 (4)	454 (5)
Mode, RT [ms]	476	617	506	567	742	638	449	501
*σ* [ms]	39 (4)	48 (8)	41 (4)	51 (7)	100 (9)	82 (7)	44 (3)	52 (4)
*τ* [ms]	173 (9)	340 (17)	144 (8)	175 (10)	177 (13)	151 (10)	82 (5)	84 (6)

**Fig. 5:**
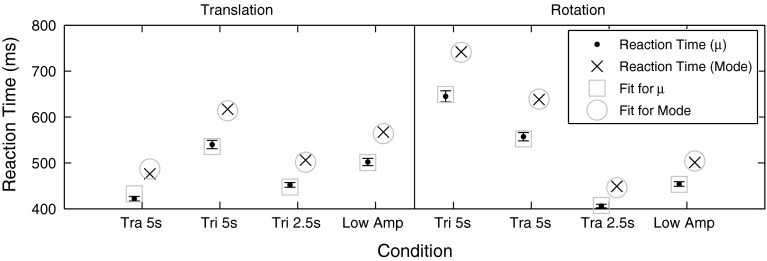
Measured RTs (based on *μ* and the mode) are shown together with the best fit for each condition. Additionally, the standard deviations of the RT estimates based on *μ* are shown. The measurements are well described by the fit

The model was fit to the measured RTs (*μ* and mode), and the resulting transfer function parameters are reported in Table 3 together with an estimate of the constant *T*_additional_ and the transfer function parameters previously obtained from threshold measurements. Using the RT estimates based on *μ*, the sum of squared errors (SSE) was 612 (ms)^2^ for translational motions and 171 (ms)^2^ for rotational motions. Using the RT estimates based on the mode, the SSE was 619 (ms)^2^ for translational motions and 80 (ms)^2^ for rotational motions. The resulting fit of the absolute RTs given by *T*_threshold_ plus *T*_additional_ is shown in Fig. 5. The mean absolute error between fit and measurements using the RT estimates based on *μ* is 5 ms for translations (5 ms using the mode) and 3 ms for rotations (2 ms using the mode).

**Table 3:** Comparison between transfer function parameters found by fitting to RT estimates based on *μ* or on the mode to parameters found in previous direction discrimination threshold studies (Soyka et al. 2011, 2012a). The parameters for translational motions are similar, whereas parameters for rotational motions show differences

	Translation				Rotation			
	*K* [s^2^/m]	*τ*_*N*_ [s]	*τ*_1_ [s]	*T*_additional_ [ms]	*K* [s^2^/°]	*τ*_*N*_ [s]	*τ*_1_ [s]	*T*_additional_ [ms]
Parameters for RT_*μ*_	1.91	4.78	0.33	290	2.86	0.054	3.65	287
Parameters for RT_mode_	2.11	4.53	0.41	339	1.01	0.006	1.04	275
Threshold studies	1.93	4.79	0.33	–	2.04	0.014	2.16	–

## Discussion

The main goal of this study was to investigate whether RTs for varying motion profiles can be described based on the same models previously used for describing threshold measurements. This would validate the modeling approach, link threshold measurements to RT measurements and thereby allow future studies to investigate self-motion perception through either RT or threshold measurements. Below we first analyze our RT findings and compare them to previous findings reported in the literature. Next, we discuss the model fit and compare the model parameters found from RT measurements to parameters found from threshold measurements. Finally, the influence of vibrations of the simulator and the values of the constants *T*_additional_ are discussed.

### Analysis of reaction time distributions

Three motion parameters (profile shape, duration and amplitude) were varied for both translational and rotational motions. It can be seen from Table 2 that all parameters influence RTs. In order to test whether the RT distributions were significantly different between conditions likelihood-ratio tests were performed (Wilks 1962). Specifically, the null hypothesis that a single ex-Gaussian distribution (3 free parameters) is sufficient to describe the data was tested against the alternative hypothesis that two ex-Gaussian distributions (6 free parameters) provide a significantly better fit. The test statistic *D* was calculated using the following formula:

4 $$ D = - 2\ln \left( {\frac{{{\text{likelihood}}\,{\text{null}}\,{\text{hypothesis}}}}{{{\text{likelihood}}\,{\text{alternative}}\,{\text{hypothesis}}}}} \right) $$

The test statistic *D* is approximately a chi-squared distribution (Wilke 1938), and if *χ* ^2^ is larger than 16.3 (inverse chi-square cumulative distribution function with 3° of freedom evaluated for* x* = 0.999), the probability p for the null hypothesis is smaller than *p* = 0.001. For example, the profile shape was varied between conditions I and II for translations (*χ* ^2^ = 460) and for V and VI for rotations (*χ* ^2^ = 127) causing a significant change in RT. The duration was varied between conditions II–III (*χ* ^2^ = 452) and VI–VII (*χ* ^2^ = 738) and the amplitude between conditions III–IV (*χ* ^2^ = 99) and VII–VIII (*χ* ^2^ = 108) also resulting in significant differences. It is well known that the duration and the amplitude of a motion influence RT, for example, Mulder’s law for rotational motions (Mulder 1908), but to our knowledge this is the first study reporting an influence of the motion profile shape on RT.

Directly comparing RTs found in this study to previous studies is difficult since RTs depend on the presented motion profile, the task (motion detection vs direction discrimination) and the measure (mean vs mode or *μ*). For example, Baxter and Travis (1938) reported a mean direction discrimination RT of 0.6 s for a 1.3 s rotational motion of 2°. Clark and Stewart (1974) reported mean direction discrimination RTs between 4.4 and 0.65 s for steps in rotational accelerations with amplitudes between 0.75 and 15 °/s^2^. Huang and Young (1981) also measured steps in rotational accelerations with amplitudes between 0.5 and 5 °/s^2^ and found mean RTs between 5 and 0.8 s. For horizontal translational motions, Arrott et al. (1990) found mean RTs between 3 and 0.7 s for steps in accelerations with amplitudes between 0.06 and 0.78 m/s^2^. Jones and Young (1978) investigated vertical translational motions and found mean RTs between 4 and 0.8 s for steps in acceleration with amplitudes between 0.1 and 0.6 m/s^2^. Note that these numbers are estimates since often results were only reported graphically. The long RTs are due to low intensity stimuli and cannot be compared to our findings. For the stronger motions, previously reported RTs are in the same range as our findings, although they seem slightly higher. This is probably due to the fact that we did not report the mean of the RTs, but the mode (and *μ*) which for skewed RT distributions is smaller than the mean (Fig. 3). These examples show that in order for RT studies to be comparable, it is important to report a description of whole RT distributions (not just the mean) and to clearly specify the presented motion stimuli.

### Model fit

From Fig. 5, it can be seen that the proposed model is able to accurately fit the RTs independent of the chosen measure (*μ* or mode). The mean absolute errors between measurements and fits are remarkably low (~5 ms) reflecting the validity of the approach. Comparing the parameters estimated based on RT measurements to the parameters previously estimated from threshold measurements (Table 3) shows a good match for the translational motion parameters. For rotational motions, the parameters differ, but are still within a reasonable range. Using the parameters obtained from RT measurements, we predicted direction discrimination thresholds and compared them to the previously measured thresholds (Soyka et al. 2011, 2012a). The root mean square error (RMS) between predictions and measurements was calculated using the parameters obtained from threshold measurements. Threshold measurements were obtained from RT using *μ* and obtained from RT measurements using the mode. For translations, it was found that the RMS based on RT measurements using *μ* was less than 1 % higher than the RMS based on threshold measurements (the RMS based on RT measurements using the mode was 27 % higher than the RMS based on threshold measurements). For rotations, the RMS based on RT measurements using *μ* was 13 % higher than the RMS based on threshold measurements (the RMS based on RT measurements using the mode was 38 % higher than the RMS based on threshold measurements). This increase in RMS for predictions based on RT measurements (compared to the RMS based on fits to the threshold data) is small and suggests that it is indeed possible to predict the previously measured direction discrimination thresholds based on the parameters obtained from RT measurements. The parameters based on the measure *μ* provide better predictions than the ones based on the mode. This represents an important finding since it indicates that the dynamics governing perceptual thresholds and RTs for self-motion stimuli are the same. This allows the use of a single model to describe both RTs and thresholds at the same time. Therefore, model parameters can be estimated based on either thresholds or RTs or even using a combination of both measures.

Note that for translational motions, the parameters based on threshold measurements were re-estimated using new IMU recordings of the stimuli tested in Soyka et al. (2011). Previously, the estimates were based on the commanded motion profiles and not on the IMU measurements of the motions. However, our motion simulator exhibits vibrations, and therefore, it is important to take these vibrations into account by using IMU measurements as described in Soyka et al. (2012a).

### Vibrations of the simulator

In the current study, deterministic vibrations of the simulator are an issue (Fig. 2). It seems that for translational motions, the vibrations are higher than for rotational motions. Note that to some extent, this is due to the fact that accelerations were measured for translational motions, whereas velocities were measured for rotational motions, and therefore, the amount of vibrations due to rotational accelerations cannot be assessed. We decided not to show numerically integrated translational accelerations or differentiated velocities, since digital filters suffer from numerical problems such as high-frequency noise for differentiation and low-frequency drift for integration.

Ideally, there should be no vibrations of a simulator during an experiment. In reality, there always are vibrations, and therefore, it is important to capture them. One advantage of our model is that it can work with arbitrary motion profiles, and therefore, with profiles recorded with an IMU that measures also the vibrations of the simulator. In this respect, the vibrations are taken into account as best as possible.

The SSE represents an indicator for the quality of the model fit. For translational motions, the SSE is higher than for rotational motions revealing a weaker model fit for translations. One of the reasons for a weak fit might be the higher amount of vibrations for translational motions. However, overall, the model fits both translations and rotations very well suggesting that the vibrations are sufficiently taken into account.

### Interpretation of the constants *T*_additional_

As discussed above, the constants *T*_additional_ that have to be added to the predictions *T*_threshold_ in order to get a description of either the parameter *μ* or the mode describing the RT distributions represent the time it takes to cognitively process sensory information and come to a decision about the direction of the motion. Using RT measurements based on the parameter *μ*, the constants for translations (290 ms) and rotations (287 ms) are rather similar. Using the mode instead of the parameter *μ*, results in clear differences between the constants (339 ms for translations and 275 ms for rotations).

We previously reported even larger differences in constants that were based on modeling using the commanded motion profiles and not the measured IMU data (Soyka et al. 2012b, c). Interpreting the differences in the constants between translational motions and rotational motions based on the modeling assumptions suggests that translations are processed slower than rotations. A possible reason for slower processing of translational motions might be that the translation signal first has to pass through a tilt-translational disambiguation mechanism before it can be detected (Angelaki et al. 1999, 2004; Merfeld et al. 1999). However, since the constants strongly depend on the measure (*μ* vs. mode) and on the fitting methodology (commanded motions vs. IMU recordings as model input), no reliable conclusions about processing differences between translations and rotations can be drawn from the constants and further research is required.

## Conclusions

In this work, we showed that RTs for varying motion stimuli can be described based on the same models used for fitting self-motion perception thresholds. This is an important finding, because it links perceptual thresholds to RTs and validates the proposed modeling approach for describing detection thresholds in our previous work. The model is based on the dynamics of the vestibular sensors and assumes a single neuronal threshold, which needs to be exceeded in order to detect motion. It provides a common basis to predict both RTs and thresholds for arbitrary motion profiles. Therefore, identifying the model based on either thresholds or RTs allows for inferring the dynamics of self-motion perception. As discussed in the introduction, RT measurements have some advantages over measuring thresholds, because they involve supra-threshold stimuli that are easier to respond to. This makes assessment of vestibular function less fatiguing for the participant and, therefore, decreases the variability of the responses. Note that due to an insufficient number of trials per participant, our analysis was performed on a group level. Further research is required to assess the performance of the proposed methodology on an individual level.

Our modeling approach allows for an accurate description of RTs in response to inertial motion stimuli and has the potential to inform recent efforts to measure the relative perceived timing of vestibular stimulation compared to the other senses (Barnett-Cowan and Harris 2009, 2011; Barnett-Cowan et al. 2010, 2012b; Sanders et al. 2011; see Barnett-Cowan 2013 for a review). For example, Sanders et al. (2011) performed temporal order and simultaneity judgments for inertial rotations paired with auditory cues. They corrected their measurements in order to take into account that a vestibular stimulus has to overcome a threshold to be perceived. Our model allows for an accurate prediction about when the sensory threshold for a vestibular stimulus is exceeded. Consequently, future research on the perceived timing of vestibular and non-vestibular cues should be guided by RT predictions based on our novel approach.
